# What is new in imaging to assist in the diagnosis of giant cell arteritis and Takayasu’s arteritis since the EULAR and ACR/VF recommendations?

**DOI:** 10.3389/fmed.2024.1495644

**Published:** 2024-10-31

**Authors:** Ruoning Ni, Minna J. Kohler

**Affiliations:** ^1^Division of Immunology, Department of Internal Medicine, University of Iowa, Iowa City, IA, United States; ^2^Department of Medicine, Division of Rheumatology, Allergy, and Immunology, Massachusetts General Hospital/Harvard Medical School, Boston, MA, United States

**Keywords:** ultrasound, vasculitis, giant cell arteritis - large-vessel, Takayasu’s arteritis, temporal arteries ultrasonography

## Abstract

Over the past decades, fundamental insights have been gained to establish the pivotal role of imaging in the diagnosis of large-vessel vasculitis, including giant cell arteritis (GCA) and Takayasu’s arteritis (TAK). A deeper comprehension of imaging modalities has prompted earlier diagnosis leading to expedited treatment for better prognosis. The European Alliance of Associations in Rheumatology (EULAR) recommended in 2023 that ultrasound should be the initial imaging test in suspected GCA, and Magnetic Resonance Imaging (MRI) remains the first-line imaging modality in suspected TAK. We summarize the recent advances in diagnostic imaging in large vessel vasculitis, highlighting use of combination imaging modalities, and discuss progress in newer imaging techniques such as contrast-enhanced ultrasound, shear wave elastography, ocular ultrasound, ultrasound biomicroscopy, integration of Positron Emission Tomography (PET) with MRI, novel tracer in PET, black blood MRI, orbital MRI, and implementation of artificial intelligence (AI) to existing imaging modalities. Our aim is to offer a perspective on ongoing advancements in imaging for the diagnosis of GCA and TAK, particularly innovative technology, which could potentially boost diagnostic precision.

## Introduction

Giant cell arteritis (GCA) and Takayasu’s arteritis (TAK) are the most common vasculitides that predominantly affect large- to medium-sized vessels (1, 2). GCA commonly affects temporal arteries, ophthalmic arteries, and vertebral arteries, known as cranial GCA, with potential complications of vision loss or ischemic stroke. GCA also involves extracranial arteries, such as subclavian and axillary arteries, known as large vessel vasculitis, associated with stenosis and aneurysm (2). TAK primarily impacts aorta and its main branches, more likely affecting subclavian, renal, mesenteric arteries (2). Early diagnosis and prompt treatment can significantly reduce the complications from vasculitis, preserve vision, and improve prognosis.

The European Alliance of Associations for Rheumatology (EULAR) updated large vessel vasculitis imaging recommendations in 2023 (3) stating that temporal and axillary artery ultrasound should be considered the first-line imaging test in all patients with suspected GCA. As an alternative to ultrasound, cranial and extracranial arteries can be examined by [^18^F]-fluorodeoxyglucose positron emission tomography (FDG-PET) or magnetic resonance imaging (MRI). For diagnosing TAK, MRI is the preferred imaging modality, with FDG-PET, computed tomography (CT), or ultrasound as alternatives. It is important to note that all imaging should be performed by a trained specialist using appropriate operational procedures and settings. Ultrasound is highly operator dependent. Generally, in the United States (U.S.), the majority of rheumatologists and radiologists have historically had little to no experience in utilizing ultrasound for diagnosing GCA, and only a few experts exist, compared to our European peers where the utilization of ultrasound for vasculitis among rheumatologists has been more accepted (4). The use of vascular ultrasound is increasingly recommended as a first-line diagnostic test for suspected GCA, and in some European institutions has replaced temporal artery biopsy (TAB) unless ultrasound findings are equivocal (3, 5). In the U.S., the preferred method for diagnosing GCA remained the TAB per 2021 American College of Rheumatology (ACR)/Vasculitis Foundation (VF) guidelines (6). As ultrasound education among U.S. rheumatologists continues to progress with the creation of the ACR Rheumatology Ultrasound (RhUS) supplemental curriculum for rheumatology fellowship training programs (7), hands-on ultrasound workshops and courses at the ACR annual meeting, and training through educational modules and Continued Medical Education (CME) courses via the Ultrasound School of North American Rheumatologists (USSONAR) (8), the utilization of ultrasound among rheumatologists in the U.S. continues to grow. And thus, the 2022 ACR/EULAR classification criteria for large vessel vasculitis now includes both TAB or a positive “halo sign” on ultrasound with equal weight scoring of 5 points toward criteria (9). Meanwhile, 2022 ACR/EULAR classification criteria for TAK emphasizes the equivalent diagnostic role of MRI, CT, ultrasound and PET on various vascular territories (10).

This review provides insights into recent advances in imaging for diagnosing GCA and TAK, including novel technology, which could potentially enhance diagnostic accuracy in clinical practice.

## Ultrasound

Ultrasound of temporal and axillary arteries is considered the first-line imaging test of choice to evaluate patients with suspected GCA per EULAR recommendations (3). Gray scale and color Doppler mode are required (3). Using the clinical diagnosis as the reference standard, pooled sensitivities and specificities of color-Doppler ultrasound (CDUS) were 75% (95% confidential interval, CI: 66–83%) and 91% (95% CI: 86–94%), respectively (11). The diagnostic accuracy has further enhanced with the advancement of ultrasound technology and improved operator expertise (12). Temporal artery ultrasound was proven to serve as a cost-effective method for diagnosing GCA accurately in patients with strong clinical suspicion, which help minimize the necessity for TAB (13). A recent study by Monjo-Henry et al. observed increased intima-media thickness (IMT) in GCA by vascular ultrasound of the carotid, subclavian and axillary arteries compared to atherosclerosis (14). Cut-off values of IMT were proposed for diagnosing GCA when compared both to clinical evaluation and MRI findings with consideration of cardiovascular risks; these await further validation (15). Schäfer et al. compared GCA patients with healthy controls and suggested cut-off values of the common superficial temporal arteries, the frontal and parietal branches and the axillary arteries are 0.42, 0.34, 0.29, and 1.0 mm, respectively (16). This led to the development of different scoring systems such as the Southend Halo Score (17) and Outcome Measures in Rheumatology (OMERACT) GCA Ultrasonography Score (OGUS) (18). Southend Halo Score and OGUS were evaluated to assess the diagnostic accuracy and showed an optimal cut-off value of 14.5 (sensitivity of 74.4% and specificity of 97.2%) and 0.81 (sensitivity of 79.07%, specificity of 97.22%), respectively (19). A reduction of the IMT of the temporal artery can be observed within 2–3 days following treatment with pulse glucocorticoids (20). Meanwhile, the normalization of the mean IMT of the axillary artery was observed after 7 days (21). This suggests that temporal artery ultrasound should be performed as soon as possible for diagnostic purposes, even though the treatment itself should not be delayed.

In addition to training the ultrasonographer on how to scan to identify anatomic vessels, it is imperative to also have high quality ultrasound equipment available within the clinic with a high frequency probe to accurately visualize and measure IMT. Without knowledge to adjust ultrasound settings appropriately, false negatives or positives can be created. A standardized training program with theoretical knowledge, reader evaluation session and hands-on scanning workshop provided excellent inter-reader and intra-reader reliability (12, 22). As more rheumatologists receive standardized vasculitis ultrasound (VUS) training to specifically evaluate for vasculitis with presence of the halo sign, and better imaging quality of ultrasound equipment becomes more accessible, the opportunity for rheumatologists to incorporate VUS into their clinical practice will gradually increase, similar to the growth of musculoskeletal ultrasound among rheumatologists.

As more imaging is obtained and the use of Artificial Intelligence (AI) is incorporated into ultrasound equipment software, it may become easier to identify the correct anatomy and findings of GCA with more confidence and accuracy. This is already being explored with a minimum resolution requirement of 224 × 224 pixels for human experts to proficiently assess VUS images (23). This discovery served as the foundation for creating an AI-powered tool to assist in classifying ultrasound images for detecting GCA. Roncato et al. created and analyzed CDUS images in GCA via a convolutional neural network and detected halo sign with a sensitivity and specificity of 60% and 90%, respectively (24). AI also holds the potential to mitigate the operator-dependent limitations by enhancing the accuracy and consistency of ultrasound evaluations and offering a supplementary perspective to the examiner during image analysis as well as potential for more accurate measurements (25).

Contrast-enhanced ultrasonography (CEUS) may play a role in evaluating disease activity in large vessel vasculitis (26, 27). CEUS with administration of sulfur hexafluoride gas stabilized by a phospholipid and palmitic acid envelope was designed to improve the visualization of vasculature (28). The contrast-formed microbubbles within the thickened artery lesions represented neovascularization. ≥ 25% increased contrasted areas of axillary/subclavian and/or carotid arteries can distinguish active and inactive GCA with a sensitivity and specificity of 91.7% and 100%, respectively (29). CEUS of carotid artery was found to detect response and relapse correlated with clinical evaluation in TAK (30, 31).

Increased arterial stiffness is associated with complications, such as hypertension and accelerated atherosclerosis in TAK, which can be detected by shear wave elastography (SWE). SWE is a non-invasive ultrasound technique that monitors and records the velocity of shear waves to assess the elasticity of blood vessels. Ucar et al. discovered that carotid artery stiffness is significantly higher in TAK along with increased IMT detected by SWE and CEUS (32).

Ultrasound biomicroscopy (UBM) of temporal artery, also known as very-high resolution ultrasound, may predict the result of TAB. The halo sign and or the intra-arterial middle reflexive filling, detected by 50–55 MHz probe on UBM, showed positive predictive value of 44.4% and negative predictive value of 100% (33). The IMT measurement by very-high resolution ultrasound was found more sensitive than conventional CDUS with maximum frequency of 22 MHz (34). UBM has been utilized to diagnose uveitis, glaucoma and cataract in ophthalmic diseases (35), which are related with glucocorticoid toxicity and can mimic as a visual disturbance in GCA.

Ocular ultrasound is currently used in the emergency medicine setting for identification of foreign body, retinal detachment (36). Clinical visual deterioration in GCA was correlated with absence of blood flow on CDUS of orbital vessels, including ophthalmic, central retinal, nasal and temporal posterior ciliary arteries (37, 38). Ocular ultrasound can also detect vitreous echoes and optic nerve sheath thickness (39). Future studies incorporating ocular ultrasound and UBM may be of interest to better understand predictive changes that may occur in GCA prior to vision loss.

## Positron emission tomography

[^18^F]-fluorodeoxyglucose positron emission tomography/computed tomography (FDG-PET/CT) has been proven to diagnose large vessel vasculitis with pooled sensitivities and specificities of 80% (95% CI: 70–97%) and 91% (95% CI: 67–98%), respectively (11). FDG-PET/CT can provide an accurate diagnosis within 3 days of initiating high-dose glucocorticoid treatment and the diagnostic sensitivity decreases after 10 days of treatment (40). A recent retrospective study revealed the utilization of ^18^F-FDG-PET/CT in diagnosing GCA with negative temporal artery biopsy (41). Positive FDG uptake at the time of diagnosis of GCA had an increased risk for thoracic aortic aneurysm, stenosis or occlusion (42, 43). Adding iodine to FDG-PET/CT imaging may serve as a potentially synergistic tool that can concurrently concentrate on both vascular inflammation and the structural status of the blood vessels in TAK (44).

Several attempts have been made to rectify the shortcomings of conventional static FDG-PET/CT. Scoring systems using FDG uptake intensity compared to liver uptake and arterial wall calcification as semi-quantitative parameters were developed to optimize the evaluation of GCA and reduce the inconsistency between different readers (45, 46). The FDG-PET/CT is mainly valid in large vessels, including aorta, axillary/subclavian arteries instead of temporal or vertebral arteries due to disparity of imaging acquisition time in large- and medium-sized vessels (47). A prolonged 5-min acquisition time may provide a higher observer agreement than a regular 2-min acquisition time in diagnosing cranial GCA, along with usage of vascular scores (48). Dynamic-whole body FDG-PET/CT was introduced to remove the radioactivity in the luminal blood pool and better distinguish the walls of vessels (47).

Integration of PET imaging with MRI provides a more accurate anatomical visualization of PET tracer uptake, especially in cranial GCA (47). PET/MRI may better define active inflammatory from inactive fibrous large vessel vasculitis in GCA and TAK compared to PET/CT and has lower radiation (49, 50).

Discovering a novel tracer in PET is also intriguing to improve diagnostic accuracy in GCA. Tissues and cells vie for the absorption of both glucose and FDG. Hyperglycemia is not uncommon in patients with suspected large vessel vasculitis on empiric high dose glucocorticoids and elevated circulating blood glucose affects the interpretation of FDG-PET (40, 51). Somatostatin receptor 2, a macrophage marker involved in the pathogenesis of GCA and TAK, showed higher uptake in active large vessel vasculitis compared to inactive vasculitis and atherosclerosis (52). Given its extremely low background noise in the brain and heart, it may permit detecting the involvement of coronary artery in TAK and intracranial artery in GCA (52). Fibroblasts are also recruited in vasculitis while radiotracers based on fibroblast activation protein inhibitor and ^68^Ga may detect active inflammation where results from ^18^F-FDG-PET/CT are not definitive (53).

AI-based segmentation of vasculature can expedite pre-analysis processing steps in PET quantification, to improve molecular and structural accuracy and enhance inter-reader reliability (54).

## Magnetic resonance imaging

Magnetic resonance imaging (MRI) of cranial arteries has a pooled sensitivity of 82% (95% CI: 76–86%) and specificity of 92% (95% CI: 84–97%) with clinical diagnosis of GCA as reference standard (11). MRI remains the preferable imaging test to investigate mural inflammation or luminal changes in patients with suspected TAK (3, 55).

3D-compressed sensing T1-weighted black blood high resolution MRI (BB-MRI) allows precise visualization of intracranial vessel wall inflammation. The involvement of intracranial arteries, including internal carotid artery, vertebral artery, posterior cerebral artery and basilar artery, was discovered by BB-MRI in GCA without artery stenosis or occlusion (56). Additional research is needed to distinguish the findings from atherosclerosis and stratify the risks of stroke in this population.

Application of orbital MRI may assist in stratifying patients with high-risk of vision loss in GCA. Gadolinium-enhancement of the optic nerve sheath and ophthalmic artery wall was found to correlate with visual symptoms and fundoscopic examinations (57, 58). Pathologic orbital MRI findings were observed in asymptomatic patients, clinically unaffected eyes or fundoscopic-negative exams (59). This may indicate early ischemic changes and possible development of posterior ischemic optic neuropathy. Further studies are required to determine the clinical significance and prognosis of abnormal subclinical orbital MRI.

## Computed tomography angiography, optic coherence tomography and fluorescein angiography

Computed tomography angiography (CTA) revealed a sensitivity of 73.3% and a specificity of 77.8% in patients with suspected GCA. These results were based on the reference standard of clinical diagnostic criteria of GCA after 6 months (60). CTA can be utilized to screen at diagnosis for aneurysm, dissection, or stenosis.

Optic Coherence Tomography (OCT) has been utilized in patients with GCA to assess ocular manifestations. OCT can detect optic disc edema, thickening of the inner retinal nerve fiber layer and ganglion cell layer, and loss of layer structure in acute stages of optic neuropathy and retinopathy. In later stages, OCT can show diffuse atrophy of the inner retina (61). Full-field OCT of TAB shows potential for identifying characteristic pathological lesions of GCA within minutes (62). OCT angiography has been used to describe chorioretinal signs in GCA, including choroidal ischemia, which is a key angiographic indicator in the diagnosis of GCA (63).

Positive fluorescein angiography or indocyanine green angiography was found with a sensitivity and specificity of 88% (95% CI: 69–97%) and 74% (95% CI: 49–91%), respectively, when compared to clinical diagnosis (64). Positive imaging tests were identified as either a delay in the filling of choroidal vessels or the existence of choroidal areas without vascularization. Due to its invasiveness, catheter-based angiography is no longer the preferred initial imaging method.

## Comparison and incorporation of multiple imaging modalities

Ultrasound of cranial and extracranial arteries showed high sensitivity and specificity to diagnose GCA compared to other imaging modalities. Adding ultrasonography of extracranial arteries to cranial arteries can increase sensitivity from 70% (95% CI: 59–79%) to 89% (95% CI: 73–96%) to detect GCA while preserve specificity around 91% (11). Extracranial involvement can be identified by both ultrasound and FDG-PET/CT (65, 66). FDG-PET/CT can detect aortitis in 33.3% of patients with positive ultrasound of extracranial arteries and 8.3% of patients with negative ultrasound findings were found with aortitis on FDG-PET/CT (67). Hemmig et al. concluded that MRI of subclavian/axillary arteries aligned with PET/CT findings but less frequent on ultrasound (68). Notably, vasculitis was defined qualitatively (69) and duration of steroid treatment varied before the imaging tests. The results of BB-MRI without contrast were consistent with FDG-PET/CT in diagnosing GCA (70). A recent nested-case control study compared CDUS, FDG-PET/CT and MRI with clinical diagnosis of GCA at 6-month follow up. CDUS had the highest sensitivity of 69.6% (95% CI: 50.4–88.8%) and equivalently high specificity among all the imaging modalities (71).

Multimodal imaging can improve diagnostic accuracy with a comprehensive assessment of both cranial and extracranial involvement (65). A diagnostic algorithm with ultrasound, MRI and retinal angiography was proposed to optimize the diagnostic performance of imaging in GCA (64). In this small sample study, it was proposed to initiate investigations with MRI, followed by ultrasound or retinal angiography to yield best diagnostic performance. This requires further validation in large populations. Multimodality imaging, including ultrasound, CT, MRI, and PET/CT, provides a more accurate and comprehensive diagnostic approach for GCA, which is essential for timely initiation of treatment to prevent serious complications. Additional studies are needed to investigate how multi-modal quantitative imaging to assess degree of disease burden may impact treatment response and relapse rates.

## Conclusion

Progressive advances in imaging technologies hold promise for improving the accurate diagnosis and monitoring of large vessel vasculitis. VUS has already shown its clinical impact on expediting large vessel vasculitis diagnoses, however further ultrasound education to teach VUS expansively is needed to make this skillset more widespread and accessible, similar to what has happened with MSKUS use among rheumatologists. Utilization of various imaging modalities including ultrasound, CT +/− PET and MRI to evaluate vasculitis both qualitatively and quantitatively will continue to assist in expedited diagnosis in conjunction with a good history and clinical exam. Additional advances in ocular and orbital imaging may also provide new insights into earlier diagnosis of disease. In cases where vasculitis is suspected but the initial imaging test is negative, combination use of imaging should be considered to obtain the optimal diagnostic accuracy for large vessel vasculitis. Despite substantial technological advancements over the past decade, the validation of new imaging modalities and standardized protocols as well as potential for the concomitant use of AI are still needed before they can be incorporated into routine clinical practice.
